# Mining for salt-tolerant genes from halophyte *Zoysia matrella* using FOX system and functional analysis of *ZmGnTL*

**DOI:** 10.3389/fpls.2022.1063436

**Published:** 2022-11-17

**Authors:** Yuying Zheng, Junqin Zong, Jun Liu, Ruying Wang, Jingbo Chen, Hailin Guo, Weiyi Kong, Jianxiu Liu, Yu Chen

**Affiliations:** ^1^ College of Agro-Grassland Science, Nanjing Agricultural University, Nanjing, China; ^2^ Institute of Botany, Jiangsu Province and Chinese Academy of Sciences, Nanjing, China; ^3^ Department of Horticulture, Oregon State University, Corvallis, OR, United States

**Keywords:** *Zoysia matrella*, FOX hunting, salt-tolerant genes, *ZmGnTL*, regulatory mechanism

## Abstract

*Zoysia matrella* is a salt-tolerant turfgrass grown in areas with high soil salinity irrigated with effluent water. Previous studies focused on explaining the regulatory mechanism of *Z. matrella* salt-tolerance at phenotypic and physiological levels. However, the molecular mechanism associated with salt tolerance of *Z. matrella* remained unclear. In this study, a high-efficient method named FOX (full-length cDNA overexpression) hunting system was used to search for salt-tolerant genes in *Z. matrella*. Eleven candidate genes, including several known or novel salt-tolerant genes involved in different metabolism pathways, were identified. These genes exhibited inducible expression under salt stress condition. Furthermore, a novel salt-inducible candidate gene *ZmGnTL* was transformed into *Arabidopsis* for functional analysis. *ZmGnTL* improved salt-tolerance through regulating ion homeostasis, reactive oxygen species scavenging, and osmotic adjustment. In summary, we demonstrated that FOX is a reliable system for discovering novel genes relevant to salt tolerance and several candidate genes were identified from *Z. matrella* that can assist molecular breeding for plant salt-tolerance improvement.

## Introduction

Soil salinization has been an adverse environmental factor restricting plant growth and development, as well as limiting plant production and quality (Katerji et al., 2003; Zhang et al., 2013; Zhang and Shi, 2013; Zhang et al., 2022). Therefore, improving plant salt-tolerance will be crucial for crop production in large saline regions. Understanding the physiological and molecular mechanisms are beneficial for plant adaption to salt stress (Van Zelm et al., 2020). Over the past two decades, scientists have described several regulatory pathways related to plant salt tolerance, including ion balance (Ji et al., 2013; Benito et al., 2014; Almeida et al., 2017), osmotic adjustment (Flowers et al., 2015; Slama et al., 2015), and reactive oxygen species (ROS) degradation (Yang and Guo, 2018a; Yang and Guo, 2018b). These pathways have been further verified through gene function analysis, such as *Salt Overly Sensitive 1-3* (*SOS1-3*) involved in Na^+^ exclusion and ion homeostasis control in many plant species (Zhu, 2000; Shi et al., 2003; Han et al., 2022), antioxidant enzymes *GhSOD1* and *GhCAT1* in cotton (Luo et al., 2013), and *P5CS* related to proline metabolism conferring salt-inducible osmotic adjustment in rice (Igarashi et al., 1997), etc. As described above, most of these salt-tolerant genes were identified from model plants or crop species, very few salt-tolerant genes have been explored from halophytes that adapt to higher salinity.

Halophytes, such as non-salt secreting type *Thellungiella halophile*, *Salicornia brachiate*, *Suaeda corniculate* (Mishra and Tanna, 2017), *Suaeda maritima* (Zhang et al., 2013) and *Puccinellia tenuiflora* (Han et al., 2022), and salt secreting type *Limonium bicolor* (Gao et al., 2021), *Avicennia officinalis* (Jyothi-Prakash et al., 2014) and *Zoysia matrella* (Chen et al., 2015), can survive from high salinity conditions with some of them even directly irrigated with saline water. Systematic screening for salt-tolerant genes from halophytes will provide valuable information for explaining the underlying molecular mechanism of their salt tolerance. Several methods such as DNA seq, RNA seq, proteomics, and metabonomics have been successfully applied for gene mining in plants (Chen et al., 2015; Yuan et al., 2015). Genes involved in the special ultrastructure of salt glands were discovered in *L. bicolor* through RNA seq method (Yuan et al., 2015), which has been a primary approach in most non-model plants. However, functional genes can change in post-transcriptional level which could not be detected by RNA seq. FOX system as a gain-of-function method using heterologous overexpression of full-length cDNA libraries in model plant *Arabidopsis* has been successfully applied for gene mining in *Arabidopsis* and rice (Ichikawa et al., 2006; Higuchi et al., 2011; Higuchi-Takeuchi and Matsui, 2014). For example, *TsHsfA1d* from *Thellungiella salsuginea* identified *via* FOX hunting system functioned as a positive regulator of heat stress response in *Arabidopsis* (Higashi et al., 2013). Overexpressing rice *OsREX1-S* screened through FOX was confirmed to enhance tolerance of host plants to cadmium (Kunihiro et al., 2014). *OsCPK21* cloned from full-length cDNA overexpressed rice was involved in the positive regulation in response to abscisic acid (ABA) and salt stress (Asano et al., 2011).

Halophyte *Zoysia matrella* is an excellent warm season turfgrass that can growth in saline soils. Our previous research mainly focused on the salt tolerance evaluation and physiological responses of *Z. matrella* to salinity. Whereas the molecular mechanism of its salt tolerance is still unclear. Moreover, we identified several potential salt-genes from *Z. matrella* through yeast-based FOX system (Chen et al., 2015). In current study, we aimed to construct the *Arabidopsis*-based FOX system for further screening candidate salt-tolerant genes for future molecular breeding. In addition, we selected a novel salt-inducible candidate gene *ZmGnTL* (β-1,6-N-acetylglucosaminyltransferase like enzyme) for functional analysis.

## Materials and methods

### Full-length cDNA expression library construction of *Z. matrella*

The cDNA entry library plasmid was produced in our previous work (Chen et al., 2015). The library plasmid was inserted into plant expression vector pEarleyGate103 with recombination reaction system (Invitrogen, USA). The reaction products were transformed into ElectroMAX™ DH10B™ T1 competent cells by electroporation, and 50 μL of 100-fold diluted cells were plated on a LB solid medium containing 50 mg L^-1^ kanamycin. Kanamycin resistant bacterial colonies were counted after 12 h and transferred to 1 mL 50 mg L^-1^ kanamycin LB liquid medium for propagation. The recombination fragment size was estimated by polymerase chain reaction (PCR) with universal primer pair (TATCCTTCGCAAGACCCTTCCTCTA/GGTAAGTTTTCCGTATGTTGCATCA) of pEarleyGate103 vector.

### Transformation of *Arabidopsis*, screening of salt-tolerant plants, and functional analysis of *ZmGnTL*

The expression library plasmid was transformed into *Agrobacterium tumefaciens EHA105* competent cells by electroporation, and the transformed cells were grown in LB solid medium containing 50 mg L^-1^ kanamycin for three days. All kanamycin resistant colonies were collected and suspended in 5% sucrose solution plus 0.5% Silwet-L77, and then introduced into *Arabidopsis thaliana* accession Columbia (Col-0) *via* floral dip method. The T1 generation seeds were obtained and screened in Murashige and Skoog (MS) solid medium containing 25 mg L^-1^ ampicillin, 20 mg L^-1^ glufosinate ammonium and 150 mM NaCl. Salt-tolerant transgenic plants were selected and transplanted into the soil. Leaves of those transgenic plants were used for extracting DNA, and PCR was performed using universal primer pair (F1/R1: TATCCTTCGCAAGACCCTTCCTCTA/GGTAAGTTTTCCGTATGTTGCATCA) of pEarleyGate103 vector. Each PCR product was sequenced and BLASTX (http://blast.ncbi.nlm.nih.gov/Blast.cgi) was performed using these DNA sequences to identify putative salt tolerant genes.

*ZmGnTL* gene was reamplified from the cDNA template of *Z. matrella* using ORF primer pair (ccggtcgacATGACGTCACCGGCGCCGGCGTACA/agtgaattcgtGTCACGTAGGATGACCGAGTCCGC) and then inserted into expression vector pEarleyGate103 with recombination reaction system. For further gene functional analysis, *ZmGnTL* was retransformed into *Arabidopsis* and the transgenic plants overexpressing *ZmGnTL* were screened following the methods of MS solid medium containing 20 mg L^-1^ glufosinate. gDNA-PCR and RT-PCR was detected using primer pair (F1/R1, shown in above). The seeds of WT and T3 generation homozygous lines were sterilized and planted in MS solid medium containing 0 or 120 mM NaCl, and the growth and biomass of seedlings were measured for salt tolerance analysis. In addition, the 18d old plants of WT and T3 generation transgenic Arabidopsis were treated with 150 mM and 200 mM NaCl in pots containing nutrient soil for 15d, and the phenotype was observed and physiological indexes were analyzed following the methods below.

### Expression analysis of candidate salt-tolerant genes

*Z. matrella* were hydroponic planted in Hoagland nutrient solution and treated with 300 mM NaCl concentration. The leaves were collected at 0, 1, 3, 6, 24, 48 h and RNA were extracted using Trizol RNA Kit (Invitrogen, USA). For the reverse transcription reaction, 0.5 μg RNA was used in the reaction with PrimeScript RT reagent Kit and treated with gDNA Eraser (TaKaRa, USA). Primer pairs of candidate salt-tolerant genes and *ZmACT* (GenBank Number: GU290545) as a reference gene are displayed in **Table 1**. LightCycler 480 SYBR I master (Roche, Switzerland) was used for each 15 μL reaction, which contained 5 μL of diluted cDNA (60 ng/μl), 7.5 μL 2×SYBR I master, 0.4 μL each primer (10 μM), and 1.7 μL ddH_2_O. The qRT-PCR reactions were performed using a LightCycler 480 II (Roche, Switzerland) with the following cycling conditions: an initial denaturation at 95°C for 10 min, followed by 40 cycles of 95°C/15 s, 58°C/15 s and 72°C/30 s, and thereafter melting curves were produced at 60-95°C. Gene relative expression levels were measured by 2^-ΔΔCt^ method. Each qRT-PCR analysis was performed in triplicate.

**Table 1:** qPCR primers for candidate salt-tolerance genes.

Salt-tolerant genes	Primer sequences 5’-3’(RT-Forward/RT-Reverse)
*ZmSAP8*	AAGGCAAATCCAGTGGTGAAG/AAAGGGAAAGGCATGGGTAAA
*ZmASR*	TTCCACGAGCACCACGAGA/CAGAGAGAAGAGGGCCAACAC
*ZmDUF1644*	GACAACGAAGAAGATGATAACCC/CCAACTCCCACACGACAGT
*ZmGnTL*	CACCCGAGTGTCTTGAGCCA/CAACCTAATAATCCCGTGTTTC
*ZmSANT*	CAAGAGAAAGCACAGAAAGAACC/CAAGGGAAACATTACAACAAGG
*ZmZAT*	CCAGTAGGCTAATCTCAGGCTTC/CAACGACAGGATAGACAGACACC
*ZmLectin*	CATGGTGGTGTGCGTGATG/AAGACAGGAGCGGGTTGGA
*ZmDBTNBT*	TCATCCTCAAGGCTCCGTT/CCTGCCGTCAATTTTTTCC
*ZmGRX*	CAAGGAAAACTGAGAGAGAGGC/AGGAACAGGGGAAACAAAGAA
*ZmUBP*	AAGGACGACCTGACAGGCAG/CGCTGTGATCCGAACCTAAAG
*ZmUAM*	GCTTGGGATGAGCTGAACCC/CCACCTGCATGACAACAGAATT

### Measurement of electrolyte leakage (EL) and relative water content (RWC)

Electrolyte leakage (EL) of leaves was measured according to the method of Blum and Ebercon (1981). Briefly, about 0.2 g fresh leaves were weighed and placed into a 50 mL centrifuge tube containing 30 mL ultrapure water. The centrifuge tubes were agitated on a shaker for 24 h at room temperature, and the initial conductivity (C_0_) was measured with conductivity meter (Thermo, New York, USA). The tubes containing the same leaf tissue samples were then autoclaved at 121°C for 15 mins and agitated for another 24 h to measure the final conductivity (C_l_). The EL was calculated as C_0_/C_l_ × l00%. About 0.2 g fresh leaves were collected to measure leaf fresh weight (FW), submerged in water for 12 h to measure leaf turgid weight (TW), then dried at 80°C for 72 h to measure leaf dry weight (DW). The RWC was calculated as (FW–DW)/(TW–DW) × 100%.

### Ion content analysis

Ten days after salt stress treatment, about 0.1 g fresh leaf or root tissue of *Arabidopsis* was oven-dried at 80°C to a constant weight. The oven-dried tissue was then decomposed for 45 min at 160°C by a microwave (ETHOS ONE, Milestone, Italy) in a digestion solution of 3 mL 65% nitric acid. After that, the liquid was diluted to 30 mL with ultrapure water. The contents of K^+^ and Na^+^ were determined by an ICP-OES (Optima 8000, Perkin Elmer, USA).

### Quantification of superoxide *O*_2_- and hydrogen peroxide (H_2_O_2_)

The $O_{2}^{-}$ production rate was measured according to Zhang et al. (2016). Briefly, leaf tissue (0.1 g) was frozen using liquid nitrogen and ground to powder, homogenized in 3 mL 65 mM phosphate buffer (PBS, pH 7.8), and centrifuged at 10000 × g for 15 min at 4°C. In the meantime, a 5:1 ratio of PBS (pH 7.8) and 10 mM hydroxylamine hydrochloride mixture was incubated at 25°C for 10 min. Then, 0.5 mL of the supernatant obtained from centrifugation was transferred to 0.5 mL mixture and incubated at 25°C for 20 min. After incubation, 1 mL 58 mM sulfonamides and 1 mL 7 mM naphthylamine were added to the mixture, and incubated at 25°C for another 20 min. Finally, 3 mL of chloroform was added to the reaction mixture, vortexed, then centrifuged at 10000 × g for 3 min. The absorbance was measured at 530 nm using a spectrophotometer (Spectronic Instruments, NY, USA). The production rate of $O_{2}^{-}$ was calculated according to the formula described by Elstner and Heupel (1976).

Each 0.5 g leaf tissue sample was frozen using liquid nitrogen and ground to powder, then 5 mL cold 0.1% trichloroacetic acid (TCA) was added and homogenized. The homogenate was centrifuged at 12000 × g for 15 min and 0.5 mL of the supernatant was mixed with 0.5 mL 10 mM PBS buffer (pH 7.0) and 1 mL 1 M KI. The mixture was incubated at 28°C for 15 min in dark. The absorbance was measured at 390 nm. The content of H_2_O_2_ was calculated based on a standard curve generated with known H_2_O_2_ concentrations.

### The analysis of enzymatic antioxidant activity

The enzymatic antioxidant activities of superoxide dismutase (SOD), peroxidase (POD), catalase (CAT), and ascorbate peroxidase (APX) were quantified using the methods described by Zhang et al. (2016). About 0.3 g leaf tissue was frozen using liquid nitrogen, ground to powder, then homogenized in 3 mL precooled 50 mM PBS (pH 7.8) containing 1% polyvinylpyrrolidone (PVP) and 0.2 mM EDTA. Homogenates were centrifuged at 15000 × g at 4°C for 20 min, and the supernatant was saved for the following enzyme activity analyses. SOD activity was measured at 560 nm absorbance. It is defined as the amount of enzyme required to cause 50% inhibition of nitroblue tetrazolium chloride reduction (Meloni et al., 2003). POD activity was measured by determining guaiacol oxidation by H_2_O_2_ at 470 nm (Lacan and Baccou, 1998). CAT activity was measured by monitoring the disappearance of H_2_O_2_ at 240 nm (Maehly and Chance, 1954). APX activity was measured by the decrease in absorbance at 290 nm for 1 min (Nakano and Asada, 1981).

### Quantification of proline, glycine betaine and soluble sugar

The proline content was measured according to the description of Abrahám et al. (2010). About 0.1 g leaf or root tissue was ground to powder using liquid nitrogen and then homogenized in 0.5 mL 3% sulfosalicylic acid. Homogenates were centrifuged at 15000 × g at room temperature for 5 min. The plant extract supernatant (100 μL) was transferred to a 1.5 mL centrifuge tube, mixed with 500 μL reaction mixture (3% sulfosalicylic acid: glacial acetic acid: Acidic ninhydrin=1: 2: 2), and incubated at 96°C for 60 min. After incubation, 1 mL toluene was added to the reaction mixture and vortexed for 20 s; the organic layer was transferred into a fresh tube after allowing the separation of the organic and water phases without disturbance for a minimum of 5 mins. The absorbance of those samples was measured at 520 nm, and proline concentration was determined by referencing to a standard curve.

The glycine betaine content was measured according to the description of Wu et al. (2018). Freeze-dried plant leaf or root (1.0 g) was ground and suspended in 25 mL 60% methanol. The extraction process was facilitated by treating the samples in an ultrasonic cleaner (Bilon, Shanghai, China) for 30 mins. Then samples were centrifuged at 15,000 × g for 8 min, and the supernatant were transferred to a new 25 mL volumetric flask and brought to volume with 60% methanol. Ten μL of each glycine betaine standard and sample was used for liquid chromatograph (Thermo fisher, Shanghai, USA) quantification at 192 nm wavelength. The glycine betaine content was determined by referencing to the standard curve.

Freeze-dried plant materials (50 mg leaf or root) were homogenized in 5 mL 80% alcohol, incubated at 30°C for 30 mins, and then centrifuged at 4500 × g at 20°C for 10 mins. The supernatant was transferred to a 50 mL centrifuge tube, mixed with 2.5 mL 80% alcohol, incubated at 30°C for 30 mins, and then centrifuged at 4500 × g at 20°C for 10 mins. This extraction step was repeated. The supernatant was transferred to a 25 mL volumetric flask and brought to volume by purified water. Then, 1 mL from the 25 mL volumetric flask was transferred to a fresh glass tube and mixed with 1 mL 23% phenol, subsequently, 5 mL 98% sulfuric acid was added and homogenized. After 15 mins, the reaction solution was cooled down to room temperature and then incubated at 30°C for 30 mins. Finally, the absorbance was measured at 490 nm, and each soluble sugar concentration was determined by referencing to its standard curve (glucose, fructose, or sucrose); and soluble sugar concentration was the sum of the three sugar contents.

## Results

### cDNA expression library

**Figure 1:**
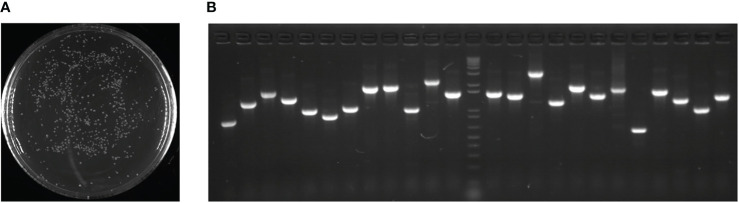
cDNA expression library quality assays. **(A)** 100-fold diluted bacteria with transformed cDNA library plasmid were cultured overnight on solid medium (LB + 50 mg L^-1^ kanamycin). **(B)** Twenty-four colonies were randomly selected for PCR to determine the size of the inserted fragment.

To evaluate the cDNA library quality, bacteria containing the library plasmid were cultured overnight on solid medium (LB + 50 mg L^-1^ kanamycin) resulting in 520 colonies on a plate (**Figure 1A**). Subsequently, 24 colonies were randomly selected for PCR to determine the inserted fragment size, and the result showed that the recombination rate was 100% and the average fragment size was 1.64 kb (**Figure 1B**).

### Salt-tolerant screening and gene mining of *Arabidopsis*

**Figure 2:**
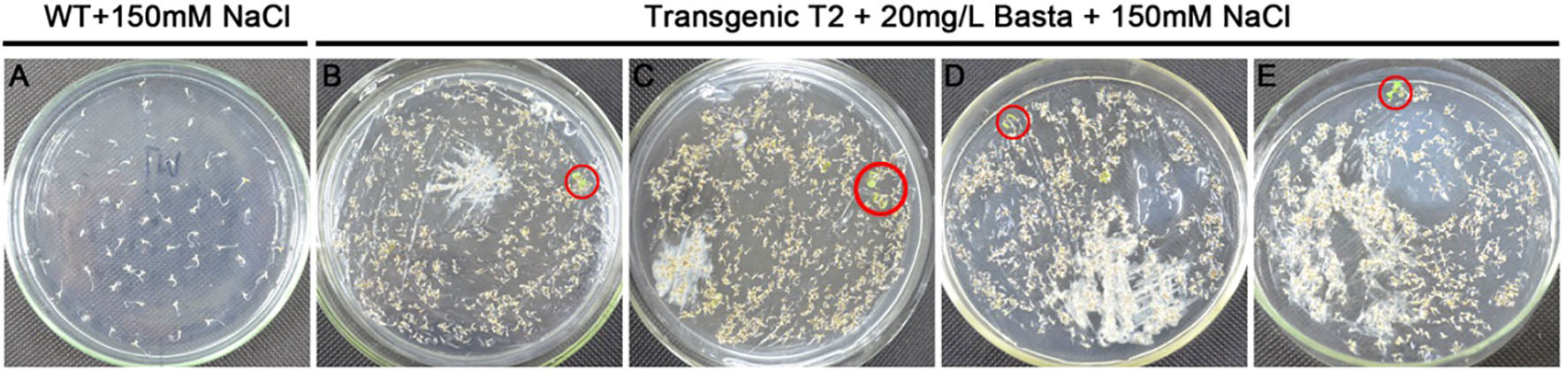
Screening of salt-tolerant *Arabidopsis* on MS plates. **(A)** Germination was completely inhibited with wildtype (WT) at 150 mM NaCl. **(B–E)** Screening transgenic lines on 20 mg L^-1^ Basta +150 mM NaCl MS plates, and salt-tolerant seedlings were circled in red.

The constructed expression library plasmid was transformed into *Arabidopsis* by floral dip method, and around 5000 T1 transgenic seedlings were obtained. The harvested seeds were used to screen salt tolerant seedlings in NaCl solid medium (MS + 20 mg L^-1^ Basta +150 mM NaCl). Finally, 25 T2 salt-tolerant plants were obtained (**Figure 2**). We extracted the DNA from these T2 transgenic lines for PCR and identified 11 candidate salt-tolerance genes by sequencing (**Table 2**).

**Table 2:** Sequence analysis and function prediction of candidate salt-tolerance genes.

Salt-tolerant genes	ORF (bp/aa)	Predicted function
FOX-ST1	516/171	A20/AN1 domain-containing stress-associated protein8(SAP8)
FOX-ST2	399/132	ABA-, stress-and fruit-ripening inducible-like protein(ASR)
FOX-ST3	1026/341	DUF1644 family Protein(DUF1644)
FOX-ST4	1089/362	GnT-Like family protein(GnTL)
FOX-ST5	975/324	SANT domain protein(SANT)
FOX-ST6	1185/395	zinc transporter(ZAT)
FOX-ST7	981/327	Ricin B-related lectin domain containing protein(Lectin)
FOX-ST8	1251/417	3-N-debenzoyl-2-deoxytaxolN-benzoyl transferase(DBTNBT)
FOX-ST9	582/194	Glutaredoxin family(GRX)
FOX-ST10	1704/568	U-box domain-containing protein 39(UBP)
FOX-ST11	1098/366	UDP-arabinopyranosemutase 1(UAM)

### Analysis of the expression of salt-tolerant genes with qRT-PCR

**Figure 3:**
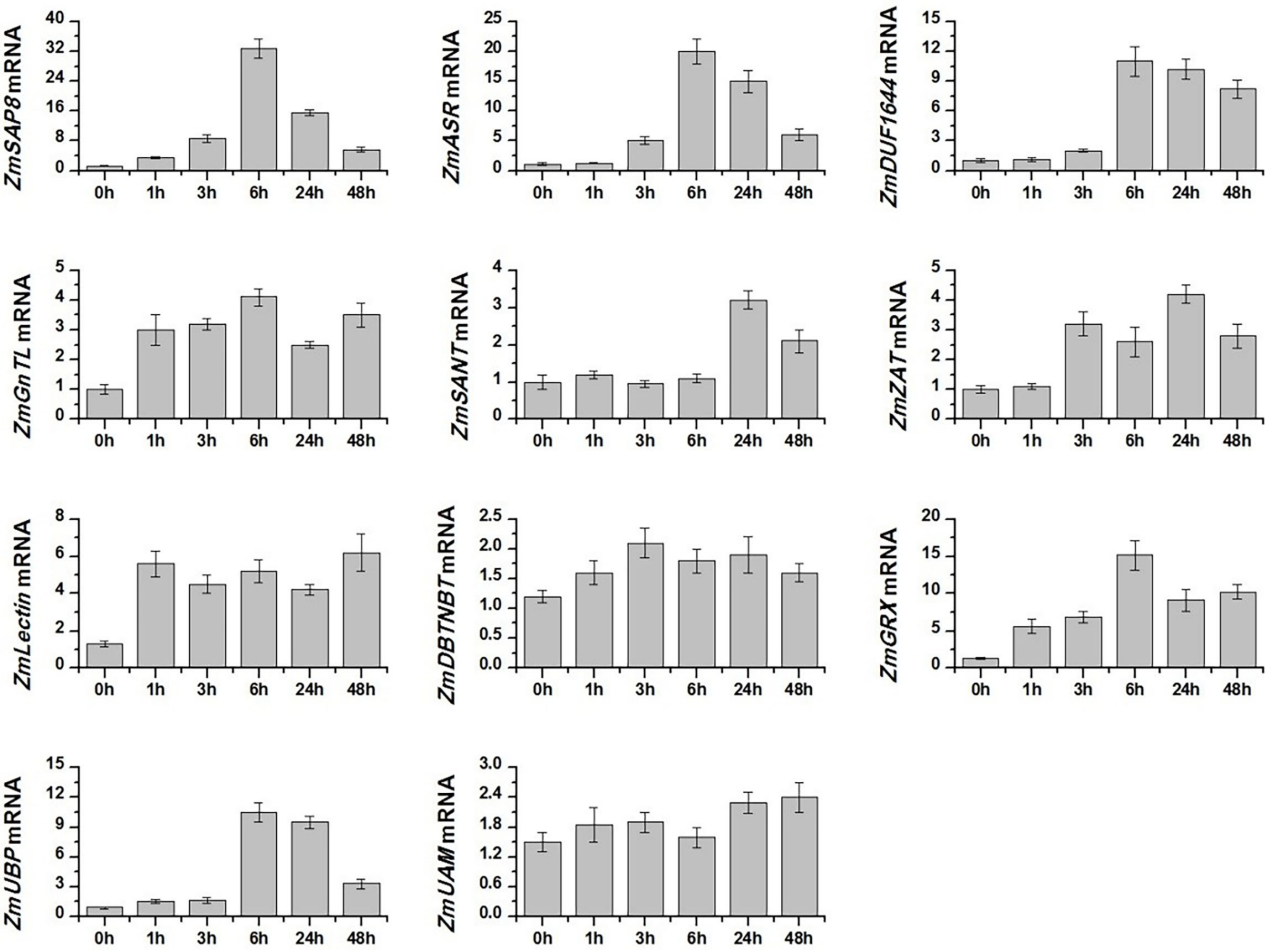
Relative expression levels of candidate salt-tolerant genes of *Zoysia matrella* treated with 300 mM NaCl. Data are shown as means ± SE of five biological replicates.

We analyzed the expression pattern of 11 candidate salt-tolerance genes response to salt stress in *Z. matrella* by qRT-PCR. The result showed that salt stress induced the gene expression of *ZmSAP8, ZmASR, ZmDUF1644, ZmGnTL, ZmSANT, ZmZAT, ZmLectin, ZmGRX*, and *ZmUBP*, whereas the expression of *ZmDBTNBT* and *ZmUAM* remained relatively stable (**Figure 3**). The expression level of *ZmSAP8, ZmASR, ZmDUF1644, ZmGnTL, ZmGRX*, and *ZmUBP* peaked at 6 h after salt stress, while the expression of *ZmSANT* and *ZmZAT* peaked at 24 h (**Figure 3**). *ZmLectin* significantly increased in expression after 1 h of salt stress, and highest expression level was observed at 48 h (**Figure 3**). Interestingly, the relative expression of *ZmGnTL* declined at 24 h after reaching the highest expression at 6 h and increased again at 48 h (**Figure 3**).

### Sequence analysis of *ZmGnTL*

**Figure 4:**
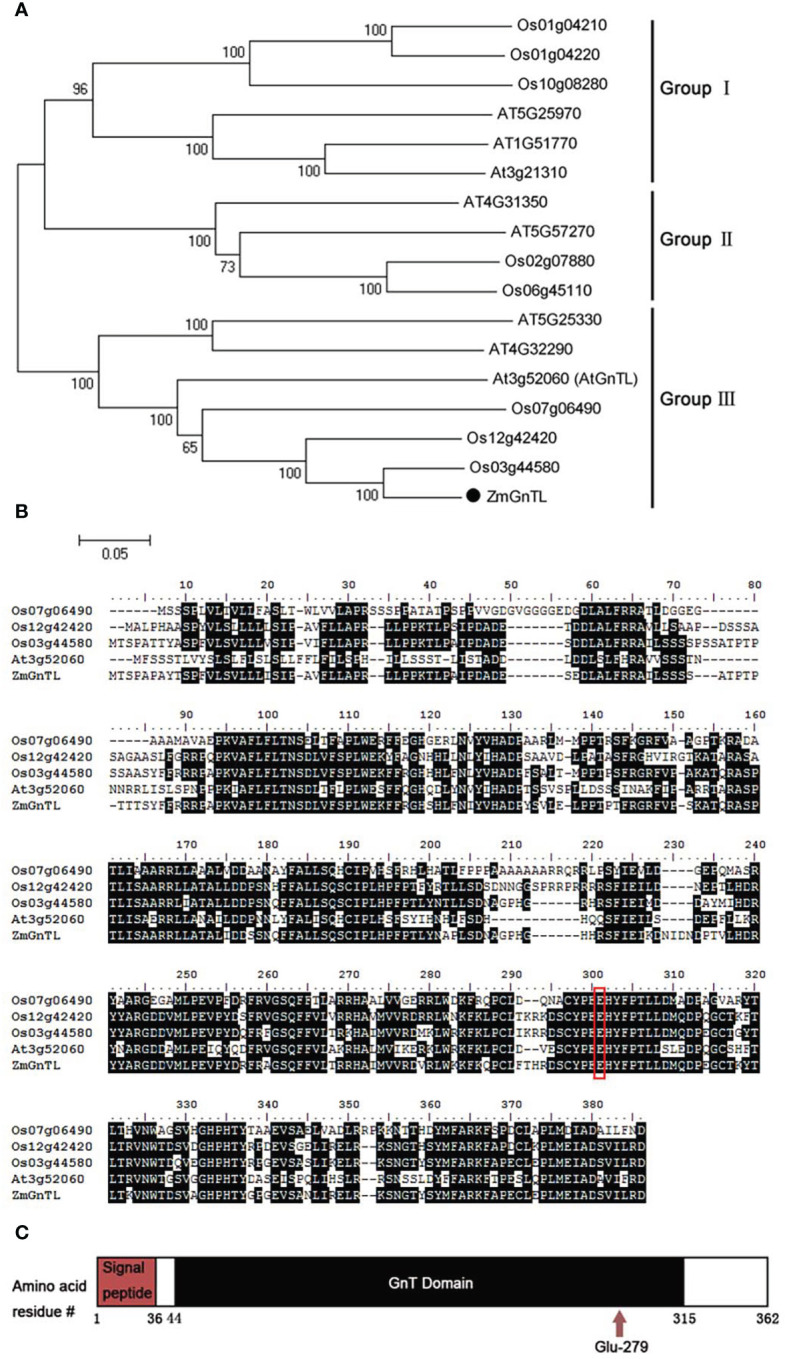
Sequence analysis of *ZmGnTL*. **(A)** Clustering analysis with related genes from rice and *Arabidopsis*. **(B)** Multiple sequence alignment analysis. **(C)** Pfam database predicted conserved domain.

To elucidate the potential role of *ZmGnTL* in the abiotic stress response of *Arabidopsis*, we cloned the sequence of *ZmGnTL*. The sequence length of *ZmGnTL* is 1089 bp, encoding 362 amino acids. Clustering analysis with related genes from rice and *Arabidopsis* suggested that those gene sequences can be divided into three groups (Group I, II, and III). In Group III, ZmGnTL was shown to have high sequence homology with Os03g44580 and AtGnTL (AT3G52060) (**Figure 4A**). A multiple sequence alignment revealed that ZmGnTL contained an amino-terminal signal peptide and a catalytic domain (GnT), which is important for glycosylation. In addition, Pfam database (http://pfam.xfam.org) analysis revealed that a conserved acid Glu^279^ site in the GnT domain may be critical for its activity (**Figures 4B, C**).

### Overexpression of *ZmGnTL* enhances salt-tolerance of *Arabidopsis*

**Figure 5:**
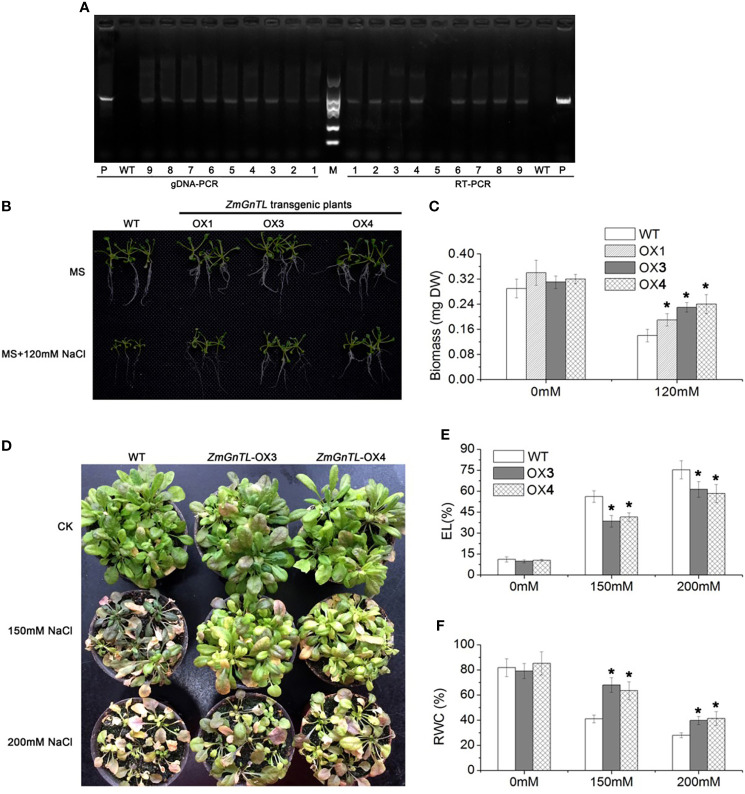
Overexpression of *ZmGnTL* enhanced the salt-tolerance in *Arabidopsis*. **(A)** gDNA -PCR and RT-PCR analysis of transgenetic lines, P (*ZmGnTL* plasmid), WT (wild type), 1-9 (transgenetic lines). **(B)** A comparison of salt-tolerant phenotypes and wildtype (WT) grown on MS medium. **(C)** Biomass of *Arabidopsis* seedlings grown on MS medium. **(D)** A comparison of salt-tolerant phenotypes and WT in soil matrix. **(E, F)** Electrolytic leakage (EL) and relative water content (RWC) of *Arabidopsis* plants. Data are shown as means ± SE of three to five biological replicates, and * indicated statistical significance at P< 0.05.

The expression of *ZmGnTL* increased substantially in *Arabidopsis* subjected to salt stress (**Figure 3**). It is plausible that *ZmGnTL* plays an important role in salt adaptation. To further confirm this hypothesis, we re-transformed *ZmGnTL* into *Arabidopsis*, and further evaluated the salt tolerance of transgenic *Arabidopsis* lines. The gDNA-PCR and RT-PCR analysis indicated that *ZmGnTL* successfully transformed into *Arabidopsis* (**Figure 5A**). The overexpressed lines (OX) under salt stress exhibited healthier phenotypes than wildtype (WT) in both MS medium and soil matrix. Whereas *ZmGnTL*-OX and WT were similar in growth under normal condition (**Figures 5B, D**). The biomass of OX lines was higher than WT under salt stress (**Figure 5C**), and the RWC content was also significantly higher in *ZmGnTL*-OX plants (**Figure 5F**). Additionally, both OX3 and OX4 exhibited lower EL levels compared with WT under salt stress (**Figure 5E**).

### The physiological changes of *ZmGnTL* transgenic *Arabidopsis* under salt stress

**Figure 6:**
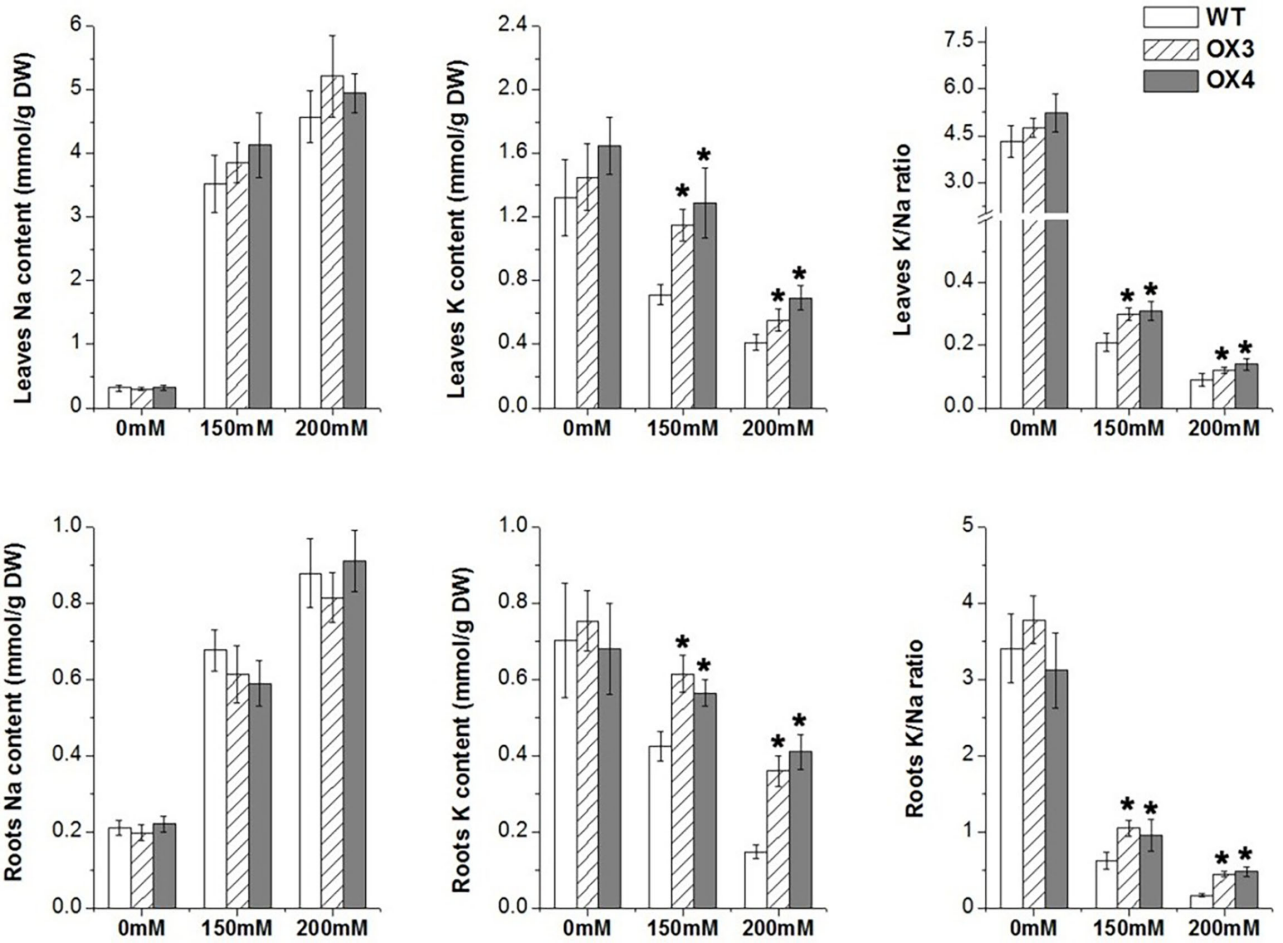
Contents of Na^+^ and K^+^ in leaves and roots of *ZmGnTL* transgenic *Arabidopsis. ZmGnTL*-OX plants and WT were treated with 0 mM, 150 mM, and 200 mM NaCl for 10 days. Data are shown as means ± SE of three to five biological replicates, and * indicated statistical significance at *P< 0.05.

To clarify the physiological changes of *ZmGnTL*-OX plants under salt stress, we measured the change of Na^+^ and K^+^ of *ZmGnTL*-OX and WT plants under different salinity treatments (0, 150, and 200 mM). Under control condition, the K^+^ and Na^+^ contents were not significantly different between *ZmGnTL*-OX lines and WT *Arabidopsis* plants. Whereas the K^+^ content was increased observably in *ZmGnTL*-OX plants, while there is no significant difference in Na^+^ content between *ZmGnTL*-OX plants and WT. (**Figure 6**). Therefore, the K^+^/Na^+^ ratio was higher in *ZmGnTL*-OX lines than that in WT under salt stress (**Figure 6**).

**Figure 7:**
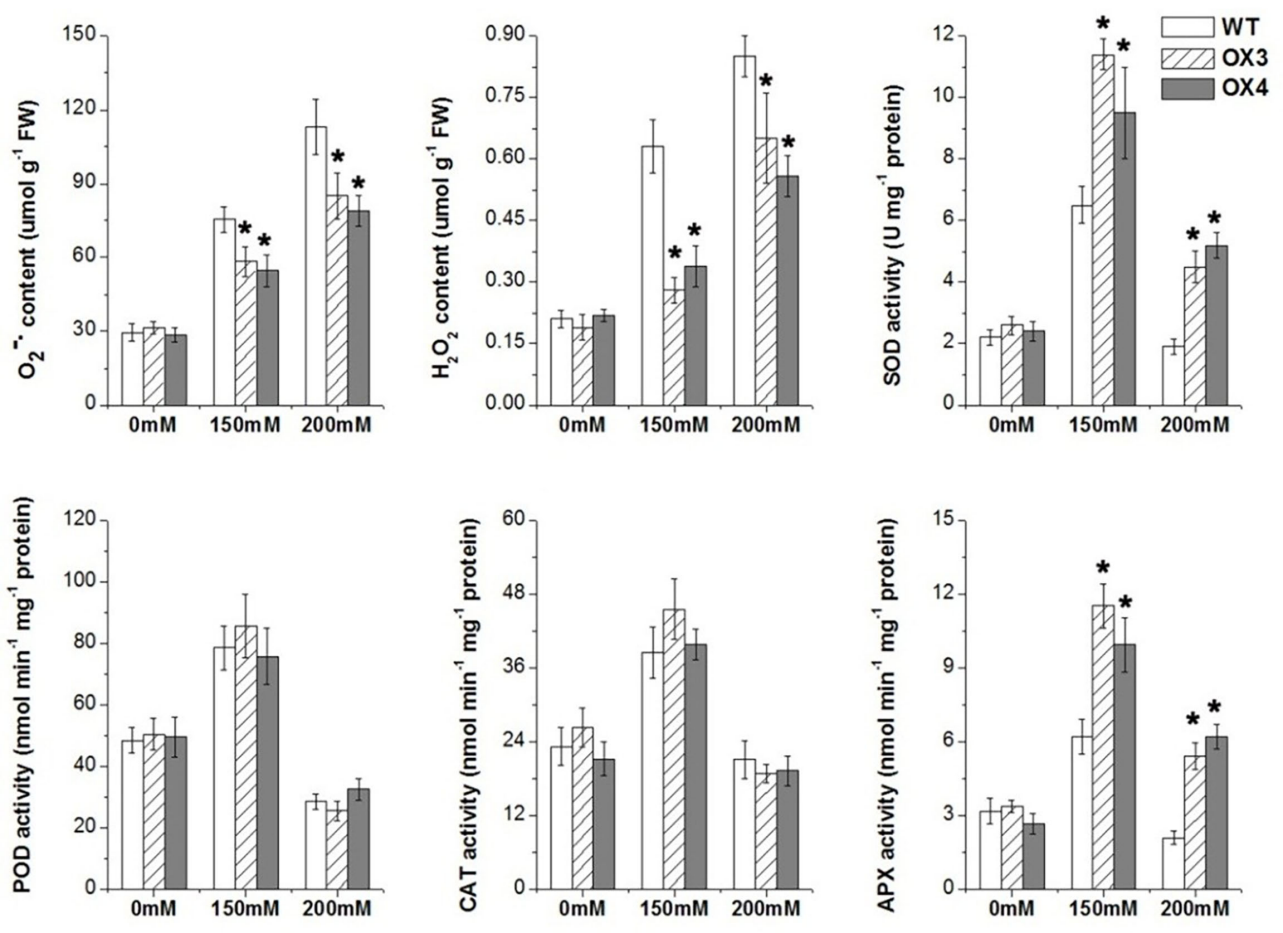
Reactive oxygen species contents and antioxidant enzyme activities of *ZmGnTL* transgenic *Arabidopsis. ZmGnTL*-OX plants and WT were treated with 0 mM, 150 mM, and 200 mM NaCl for 10 days. Data are shown as means ± SE of three to five biological replicates, and * indicated statistical significance at *P< 0.05.

**Figure 8:**
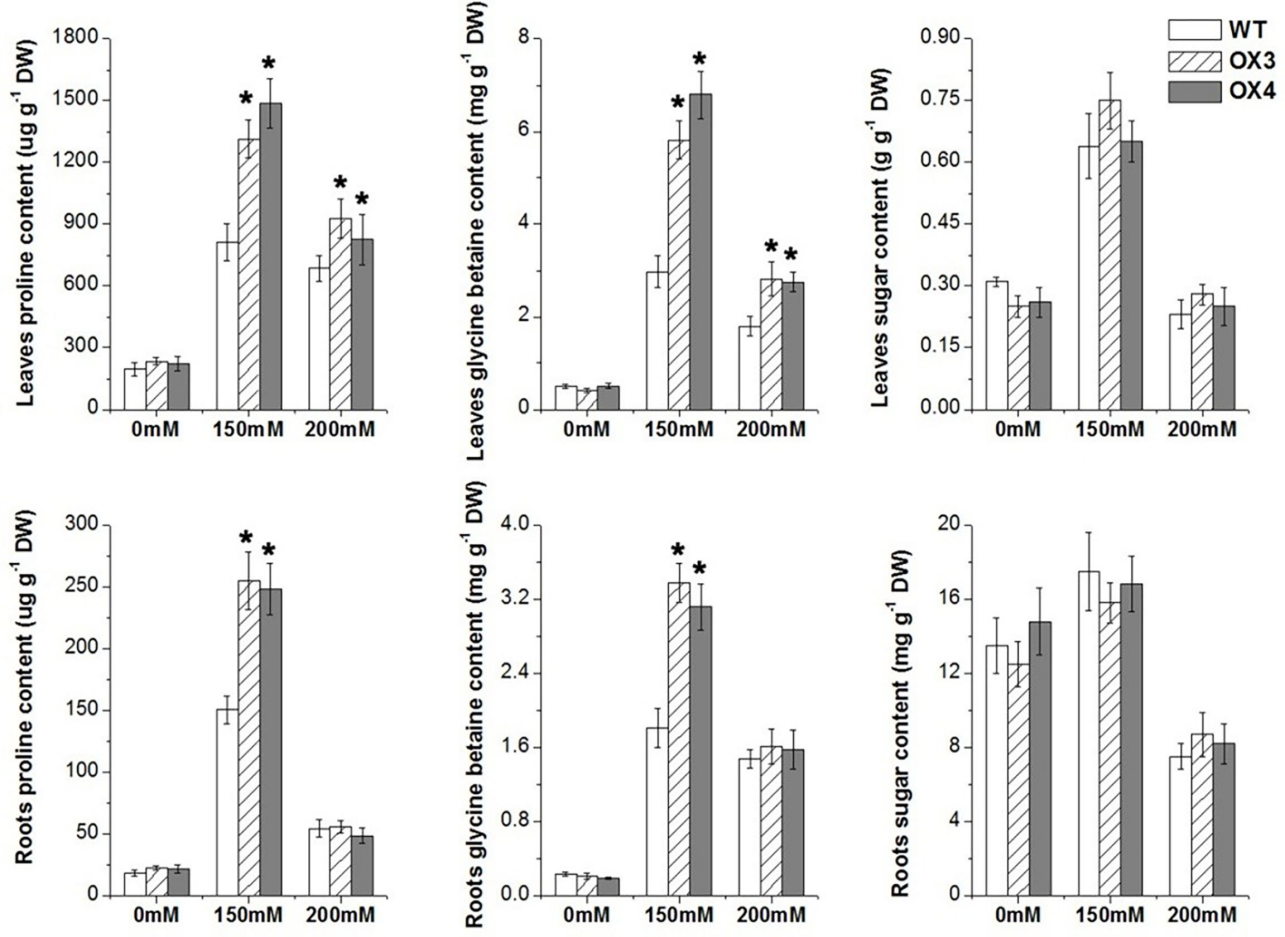
Osmolyte contents in leaves and roots of *ZmGnTL* transgenic *Arabidopsis*. *ZmGnTL*-OX plants and WT were treated with 0 mM, 150 mM, and 200 mM NaCl for 10 days. Data are show as means ± SE of three to five biological replicates, and * indicated statistical significance at *P< 0.05.

We also analyzed the change of antioxidation system and osmolytes of *ZmGnTL*-OX and WT plants under salt stress. The result showed that the overexpression of *ZmGnTL* significantly decreased the contents of $O_{2}^{-}$ and H_2_O_2_ under salt stress (**Figure 7**). The enzyme activities of SOD, POD, CAT and APX were not different between *ZmGnTL*-OX lines and WT under normal growth condition (**Figure 7**). However, the activity levels of antioxidant enzymes SOD and APX were substantially higher in *ZmGnTL*-OX lines than those in WT *Arabidopsis* under salt stress, although the activities of POD and CAT were not affected by the overexpression (**Figure 7**). Proline and glycine betaine contents were also not significantly different in roots and leaves between *ZmGnTL*-OX lines and WT under normal condition (**Figure 8**). On the contrary, the accumulation of proline and glycine betaine was increased in leaves of *ZmGnTL*-OX plants under both 150 and 200 mM NaCl compared with WT. The contents of proline and glycine in root were significantly different under 150 mM NaCl but not under 200 mM NaCl (**Figure 8**). There was not significantly difference in soluble sugar contents in both roots and leaves between *ZmGnTL*-OX lines and WT under both normal and salt stress conditions (**Figure 8**).

### *ZmGnTL* influenced the expression of salt tolerant genes

**Figure 9:**
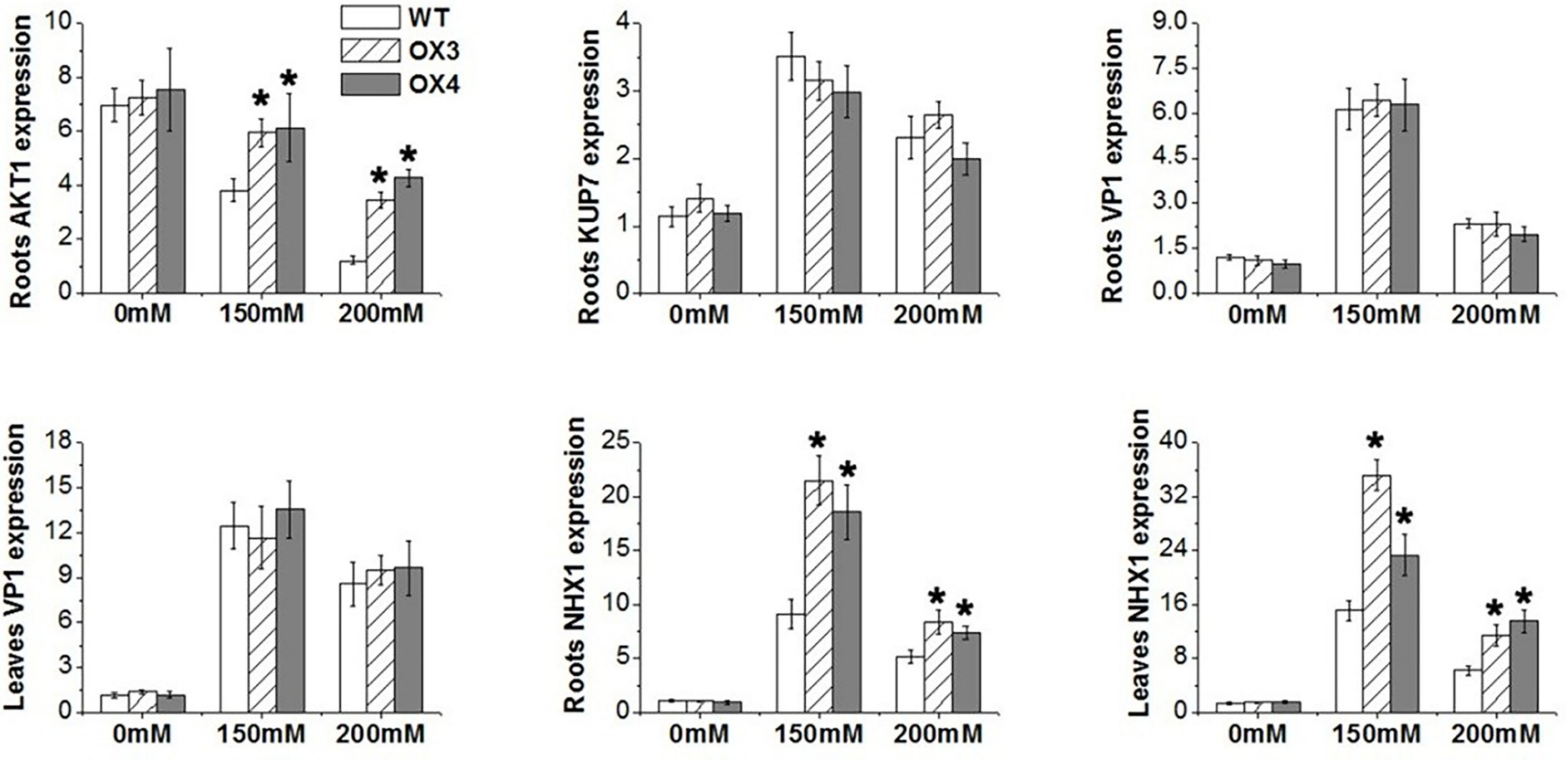
The relative expression of genes regulating ions balance in *Arabidopsis* under salt stress. *ZmGnTL*-OX plants and WT were treated with 0 mM, 150 mM, and 200 mM NaCl for 48 h. Data are shown as means ± SE of five biological replicates, and * indicated statistical significance at *P< 0.05.

**Figure 10:**
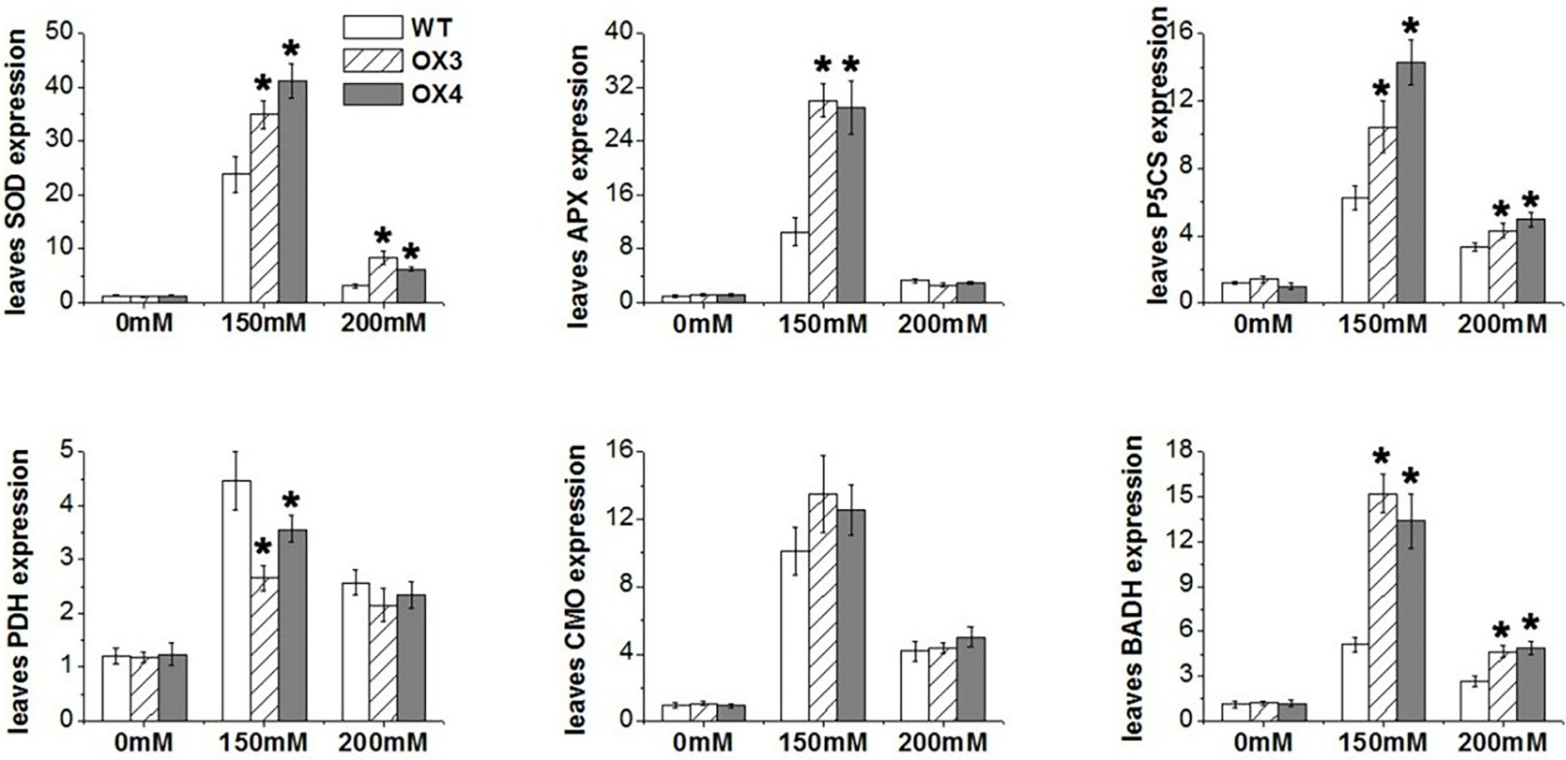
The relative expression of genes regulating antioxidant enzyme and osmolytes in *Arabidopsis* under salt stress. *ZmGnTL*-OX plants and WT were treated with 0 mM, 150 mM, and 200 mM NaCl for 48 h. Data are shown as means ± SE of five biological replicates, and * indicated statistical significance at *P< 0.05.

To explore the molecular mechanism of *ZmGnTL* in regulating salt stress, WT and *ZmGnTL*-OX *Arabidopsis* plants were subjected to 24 h of salt stress to analyze the expression of genes associated with ion transport (*AKT1, NHX1, VP1*, and *KUP7*) (Hirsch et al., 1998; Apse et al., 2003; Li et al., 2005; Han et al., 2016), antioxidation (*APX1* and *Mn-SOD*) (Li et al., 2019; Chen et al., 2022), and osmotic adjustment (*PDH, P5CS, CMO*, and *BADH*) (Peng et al., 1996; Strizhov et al., 1997; Fitzgerald et al., 2009; Luo et al., 2012). The expression of *AtNHX1* was significantly higher in *ZmGnTL*-OX lines than that in WT roots and leaves under salt stress, while the expression was similar under normal condition (**Figure 9**). In the roots, the expression of *AtAKT1* was significantly higher in *ZmGnTL*-OX lines compared with that in WT under salt stress (**Figure 9**). Overexpression of *ZmGnTL* increased the expression of *AtAPX1* under 150 mM NaCl and *AtMn-SOD* under both 150 and 200 mM NaCl conditions, but their expression levels were lower and similar between *ZmGnTL*-OX lines and WT under normal condition (**Figure 10**). Under salt stress, genes related to osmotic stress regulation, such as *AtP5CS* and *AtBADH*, were also up-regulated in *ZmGnTL*-OX transgenic lines, the expression of *AtPDH* was similar between *ZmGnTL*-OX lines and WT under both 0 and 200 mM NaCl conditions but significantly lower in *ZmGnTL*-OX lines under 100 mM NaCl (**Figure 10**).

## Discussion

### Candidate salt-tolerant genes from *Z. matrella*

Salt stress leads to various physiological and molecular changes and impedes plant growth. To alleviate the damage of high concentrations of sodium, many genes are involved in regulating the salt stress under various mechanisms. In this study, 11 salt-tolerant genes from *Z. matrella* were identified. Many of these genes have been reported in various important biological processes.

The stress-associated protein 8 (SAP8) was reported as an osmotic stress-responsive transcription factor (Kanneganti and Gupta, 2008). Previous research showed that several SAP proteins are regulated by drought and salinity stress (Kanneganti and Gupta, 2008). Another gene ASR was associated with ABA in regulating stress and fruit ripening (Yoon et al., 2021); this gene family has been identified to responding to abiotic stresses and ABA in maize and rice but is absent in *Arabidopsis* (Zhang et al., 2015; Yoon et al., 2021). For example, a study has demonstrated that overexpression of *OsASR1* and *OsASR3* improved drought and salinity tolerance in transgenic rice (Joo et al., 2013). Glutaredoxins (GRXs) are small disulfide oxidoreductases that catalyze the reduction of disulfide bridges (Rouhier et al., 2008). Studies on the function of GRXs in plants have mainly focused on model plants, and it has been shown that GRXs are involved in the stress response and hormone signaling (Morita et al., 2015; El-Kereamy et al., 2015; Verma et al., 2016; Li et al., 2021). Zinc transporter (ZAT) is a type of zinc finger proteins (ZFPs) which are transcriptional regulators in plants (Han et al., 2020). In rice, ZAT was reported to regulate the expression of several genes that involved in ROS signaling when plants were under salt stress (Sun et al., 2010). Recently, *GhZAT34* and *GhZAT79* genes from *Gossypium hirsutum* were found to enhance salt tolerance in *Arabidopsis* and cotton (Rehman et al., 2021).

Lectins are a group of structurally diverse proteins which are defined as carbohydrate binding proteins and further divided into 25 subfamilies. Lectin plays an important role in response to abiotic or biotic stimuli (Vierbuchen, 1991; Naithani et al., 2021). In this study, we also identified a Glycosyltransferases (GTs) family gene *GnTL* in *Z. matrella*. GTs family protein is required for protein glycosylation (Vogt and Jones, 2000), and studies have shown that overexpressing genes (UGT85A5 and UGT87A2) from this family increased salt-tolerance in *Arabidopsis* and tobacco (Sun et al., 2013; Li et al., 2017). The protein encoded in *Arabidopsis AtGnTL* (AT3G52060) is involved in plasmodesmata interaction (Zalepa-King and Citovsky, 2013), but the function of *AtGnTL* related to salt tolerance has not been fully described.

Using the FOX system, we also identified an interesting gene, *DUF1644*, which belong to DUFs (domains of unknown functions) families (Bateman et al., 2010). The DUF1644 gene family is highly conserved in plants, but the biological function is unclear. Recently, a salt-induced gene, *OsSIDP366* (stress induced DUF1644 family protein), was found in rice, and overexpressing *OsSIDP366* significantly improved the salt tolerance of rice (Guo et al., 2016). Additionally, we identified *ZmSANT* gene. SANT domain protein was reported to be associated with chromatin remodeling, histone acetylation and deacetylation, but the biological function is unknown (Boyer et al., 2004; Marcum and Radhakrishnan, 2019). Fox system is reliable and efficient for screening up-regulated genes under salt stress or other abiotic stresses. Using this method, we successfully identified many candidate genes for further studying the mechanism of salt tolerance in halophytes.

### Overexpression of *ZmGnTL* improved the salt tolerance of *Arabidopsis*

GnTL genes belong to the glycosyltransferase superfamily and are crucial in glycan synthesis (Fukuda and Hindsgaul, 1994) by adding the oligosaccharide side chains to glycoproteins (Siddiqui et al., 2005). This protein has been found in more than 19 plant species (Zalepa-King and Citovsky, 2013). However, many of the GnTL genes are not clearly characterized in their functions. In this study, we identified a gene *ZmGnTL* using a plant cDNA library screening method (FOX) under salt stress. Furthermore, we demonstrated the function of *ZmGnTL* in *Arabidopsis* salt tolerance. Salt stress severely inhibited the growth of *Arabidopsis*. The overexpression of *ZmGnTL* alleviated the damage of salt stress (**Figure 5C**), and the transgenic plants showed greater seedlings biomass and RWC content under salt treatment than WT (**Figures 5B, E**). Based on the data from the current study, we did not observe any advantages of the transgenic *Arabidopsis* lines overexpressing *ZmGnTL* growing under non-stress condition; those transgenic plants showed normal growth and similar genotypes as WT under non-salt stress condition, which was further supported by our results of similar physiological and gene expression measurements.

Under salt stress, higher Na^+^ accumulation in plant leads to the disruption of ion homeostasis. Since excessive Na^+^ often leads to K^+^ deficiency, plants need to modulate the Na^+^/K^+^ homeostasis through maintaining high K^+^/Na^+^ ratio (Park et al., 2016). The potassium transporters, such as the inward-rectifier K^+^ channel *Arabidopsis* K transporter (AKT1) plays an important role in K^+^ uptake and transport in the root (Nieves-Cordones et al., 2014). In this study, we found that overexpression of *ZmGnTL* increased K^+^ content and K^+^/Na^+^ ratio (**Figure 6**) through up-regulating the expression of *AtAKT1* and *AtNHX1* (a Na^+^/H^+^ antiporter, which transports Na^+^ away from the cytosol to vacuolar) under salt stress to maintain the ion balance (**Figure 9**).

Under salt stress, ion imbalance and water deficiency in the plant cell cause osmotic stress. Salt stress induces the reduction in cell turgor pressure, shrinkage of the plasma membrane, and physical alteration of the cell wall (Park et al., 2016). In order to alleviate the damage of osmotic stress, plants activate the osmolyte (such as proline, polyols, and sugars) accumulation under salt stress (Yang and Guo, 2018a). Our results demonstrated that overexpression of *ZmGnTL* increased proline and glycine betaine contents in response to salt stress (**Figure 8**). In addition, gene expression experiment also revealed that the increased expression of *AtP5CS* (proline biosynthesis) and *AtBADH* (betaine aldehyde dehydrogenase) in transgenic lines compared with WT under salt stress; however, the expression of *AtPDH* was decreased (**Figure 10**). Proline dehydrogenase (PDH) is functioned to remove free proline and prevent excessive proline accumulation after salt stress. A reciprocal regulation of P5CS and PDH was previously described to control the levels of proline during and after osmotic stress (Peng et al., 1996).

In plants, salt-stress-triggered ion stress and osmotic stress cause metabolism imbalance and toxic accumulation of ROS, which lends to oxidative damages (Yang and Guo, 2018b). Plant cells sense the accumulated ROS and respond rapidly by using regulatory mechanisms to scavenge ROS and activate a series of downstream adaptive responses (Park et al., 2016; Van Zelm et al., 2020). Several studies have shown that the activities of ROS scavenging enzymes and antioxidants are triggered by salt stress stimuli. For example, the APX and CAT are activated by salt stress to alleviate oxidative stresses (Sofo et al., 2015; Choudhury et al., 2017). In our study, the content of ROS $O_{2}^{-}$ and H_2_O_2_ were significantly lower in transgenic *Arabidopsis* compared to WT. These findings were also supported by the increased expression of SOD and APX genes in synthesizing ROS detoxifying proteins under salt stress (**Figure 6**). Therefore, our results indicated that *ZmGnTL* could effectively increase the content of antioxidant enzymes and help plants to alleviate the ROS toxicity caused by salt stress.

## Conclusion

**Figure 11:**
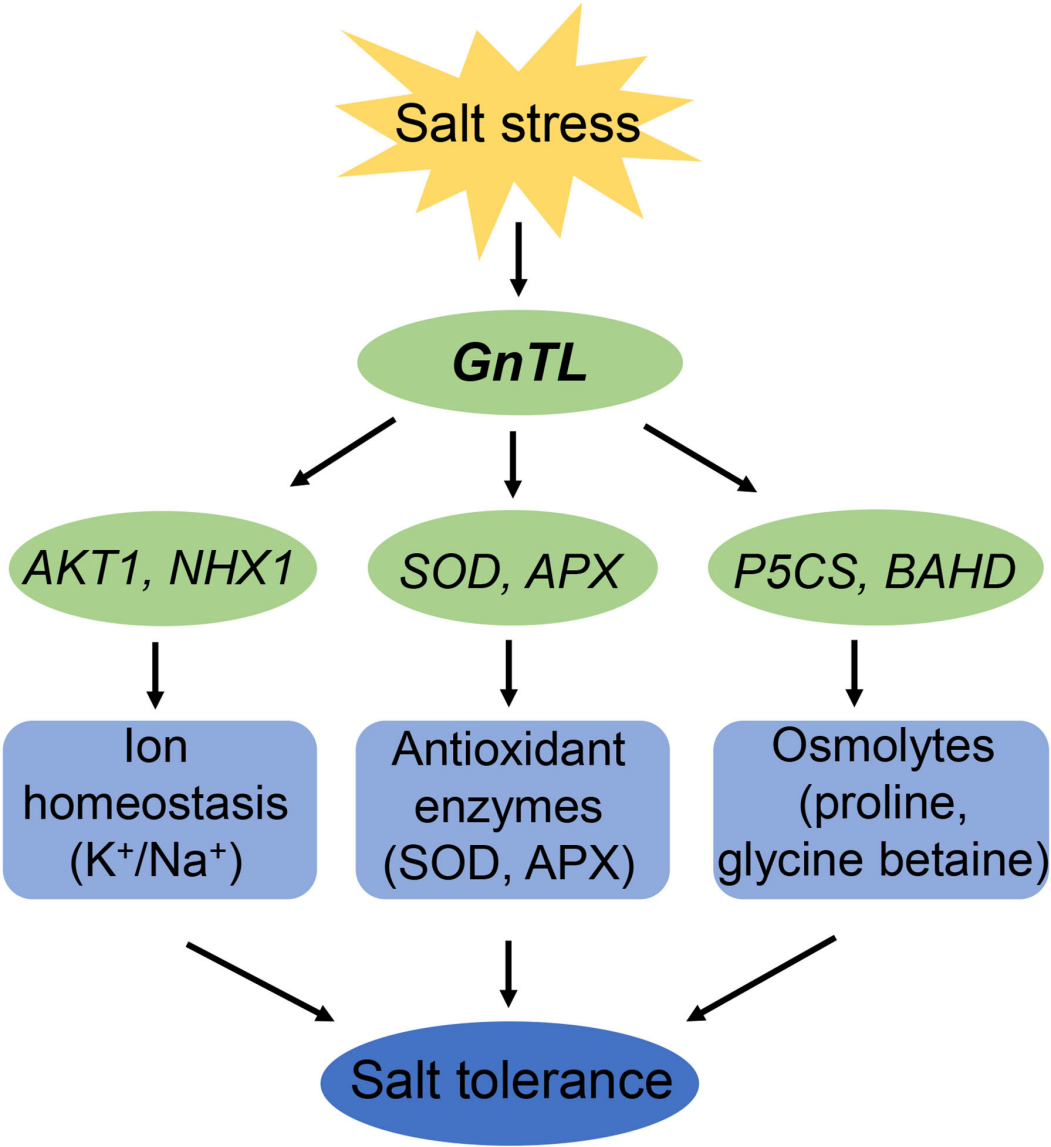
Proposed model for functional process of *GnTL* in improving plant salt tolerance.

In summary, 11 new salt tolerance candidate genes from *Z. matrella* were identified by FOX system. Among those genes, we analyzed the function of *ZmGnTL* in *Arabidopsis* in response to salt stress. Overexpression of *ZmGnTL* significantly up-regulated the expression of K^+^ transporter gene, *AKT1*, tonoplast Na^+^/H^+^ antiporter gene, *NHX1*, *SOD*, *APX*, *P5CS*, and *BADH*, and down-regulated the expression of proline dehydrogenase gene, *PDH*. Our results suggested that *ZmGnTL* was involved in alleviating ion toxicity, and oxidative and osmotic stress under salt stress (**Figure 11**). *ZmGnTL* could be an important target gene for improving crop salt tolerance through genetic engineering.

## Data availability statement

The original contributions presented in the study are included in the article/supplementary material. Further inquiries can be directed to the corresponding author.

## Author contributions

YC, JiL, and JZ designed the experiments. YZ, JZ, and JuL performed the experiments. YZ, YC, and WK analyzed the data with suggestions by JC, HG, and JuL. YZ, YC, and RW wrote the manuscript. All authors contributed to the article and approved the submitted version.

## Funding

This work was supported by the National Natural Science Foundation of China (31301806, 31872953 and 31672193).

## Conflict of interest

The authors declare that the research was conducted in the absence of any commercial or financial relationships that could be construed as a potential conflict of interest.

## Publisher’s note

All claims expressed in this article are solely those of the authors and do not necessarily represent those of their affiliated organizations, or those of the publisher, the editors and the reviewers. Any product that may be evaluated in this article, or claim that may be made by its manufacturer, is not guaranteed or endorsed by the publisher.
